# The relationship between physical activity and the health of primary and secondary school teachers: the chain mediating effects of body image and self-efficacy

**DOI:** 10.1186/s12889-024-17914-2

**Published:** 2024-02-22

**Authors:** Xiaofeng Gao, Meichao Cheng, Rong Zhang

**Affiliations:** 1 Physical Health and Art Education Research Center, China National Academy of Educational Sciences, Beijing, China; 2 School of Physical Education, Shandong University, Jinan, Shandong China; 3 Department of Sport and Leisure Studies, Namseoul University, Cheonan, Korea

**Keywords:** Active health, Physical activity, Primary and secondary school teachers' health, Body image, Self-efficacy

## Abstract

**Background:**

Active health is a new concept, model, and system to maintain the state of whole-person health. In the context of the increasingly serious health problems of primary and secondary school teachers, it is of great significance to explore the relationship between physical activity and primary and secondary school teachers’ health based on the active health perspective.

**Methods:**

The survey involving 741 primary and secondary school teachers across representative provinces in China utilized the International Physical Activity Scale, Body Imagery State Scale, and General Self-Efficacy Scale. Data analysis employed SPSS 25.0 and Amos 24.0 software.

**Results:**

While no significant gender disparities were observed in body image and self-efficacy, age groups exhibited a bipartite and “V” shaped distribution. Female teachers demonstrated higher physical activity levels (2456.46) and superior physical fitness compared to males (2297.86). A positive correlation emerged between physical activity, body image, self-efficacy, and health status. Importantly, body image and self-efficacy partially mediated the relationship between physical activity and health status, accounting for 82.31% of the total effect.

**Conclusions:**

Primary and secondary school teachers have real problems such as teachers’ physical activity is generally insufficient, teachers’ body image status is generally poor, and teachers’ self-efficacy is low; physical activity is an important factor in promoting primary and secondary school teachers’ health status, and low, medium, and high levels of activity all have a promoting effect on the health of primary and secondary school teachers, and the more active primary and secondary school teachers are in terms of physical activity, the better their body image, self-efficacy, and health status are, and the more active primary and secondary school teachers are in terms of physical activity, the more positive the physical activity, self-efficacy, and health status are. The more physically active primary and secondary school teachers are, the better their physical intention, self-efficacy and health status are.

**Suggestions:**

improve the quality and effectiveness, promote the concept of active health among teachers; empower teachers, strengthen the institutional protection of teachers’ health; reduce the burden and increase the quantity,and optimise the supply of health services for teachers.

**Supplementary Information:**

The online version contains supplementary material available at 10.1186/s12889-024-17914-2.

## Background

Education is the key to the plan for the next hundred years. The fundamental, pioneering, and overall status of education in the construction of socialist modernization has continued to deepen, making significant contributions to the development and progress of the economy, society, culture and other areas. The report of the Twentieth National Congress of the Communist Party of China (CPC) made the deployment of “accelerating the construction of a strong educational country”, which points out the direction of the development of education in the coming period and provides a guideline.

Teachers are the key to education. Primary and secondary school teachers, as the basic and strategic support for the development of China’s education, work on the front line of education and teaching for a long time, and their health directly affects the process of China’s education modernization. In recent years, with the deepening of the comprehensive reform of teacher management and the in-depth implementation of the “double-decrease” policy, the status and treatment of teachers have been improved [1], and the burden of students’ schoolwork has also been reduced, but because the teaching task of teachers has not been reduced, the quality of teaching has not been lowered, and the parents’ expectations have not been lowered, the work of primary and middle school teachers has been put forward with higher requirements, and this will inevitably lead to a change in their health. higher requirements, which is bound to seriously affect the lifestyle and health of primary and secondary school teachers. In addition, under the assessment criteria dominated by teaching performance, the teaching pressure and work intensity of primary and secondary school teachers have gradually increased, and some primary and secondary school teachers have developed such bad habits as smoking and alcoholism [2], which, coupled with the heavy teaching load, has further compressed the time for physical activities of primary and secondary school teachers, causing some primary and secondary school teachers to develop different degrees of psychological illnesses, such as depression and anxiety, and even high blood pressure, Coronary heart disease, obesity and other chronic diseases.In summary, the academic community has paid little attention to the health of the primary and secondary school teacher population, with existing research focusing on sociological and medical areas such as burnout, health inequalities [3], and unhealthy lifestyles, and relatively neglecting the constraints of teachers’ self-efficacy, body intention, and other own psychological factors on their health levels. The study helps to fill the knowledge gap in the existing literature on the relationship between health and physical activity among primary and secondary school teachers [4], providing a foundation for future research. By gaining a deeper understanding of this relationship, researchers can provide theoretical and empirical support to promote further academic exploration and practice in related fields, thereby advancing the field as a whole [5]. Overall, research on the relationship between physical activity and the health of primary and secondary school teachers can help to promote the health of the teaching population and improve their job satisfaction and overall performance [6], which in turn will have a positive impact on the development of the education system and students.

Therefore, from an active health perspective, this paper explores whether, what kind of, and how physical activity impacts on the health of primary and secondary school teachers [7], and what role psychological factors such as self-efficacy and body intention play in this to be further recognised.

Physical activity, as a major form of active health intervention, plays an important role in meeting the health needs of primary and secondary school teachers to “get less sick”, “not get sick” and “cure the disease before it occurs”. From the physiological level, physical activity can improve physical fitness and change body composition (fat loss and muscle gain); from the psychological level, physical activity is also regarded as “a good medicine for mental health” [8], which helps to enhance the individual’s sense of acquisition, self-esteem and self-confidence, as well as help to construct a positive body image [9]. Accordingly, the hypothesis was formulated: H1: Physical activity has a positive effect on the health of primary and secondary school teachers.

Body imagery refers to perceptions and attitudes towards an individual’s body, which encompasses both perceptions, emotions, cognitions, and behaviors towards the body [10], as well as social representational implications such as social norms, cultural metaphors, and symbolic values [11]. Changes in body imagery can cause individuals to be highly concerned about their weight and size, determining how they perceive their bodies. Some studies have shown that negative body imagery can seriously affect an individual’s quality of life [12], and is prone to health-hazardous behaviors such as physical anxiety, depression, and eating disorders [13]. Physical activity can enhance an individual’s body imagery, and in terms of gender differences, women are more concerned about body imagery compared to men [14]. However, it has also been suggested that the level of dissatisfaction with body imagery is comparable between men and women, with only different manifestations and focus of attention [15]. Based on the health behaviour theory, which states that an individual’s health behaviour is determined by a combination of their intentions and behavioral control factors [16], it can be inferred that the level of body image of primary and secondary school teachers may affect their attitudes and motivation for engaging in physical activity, which in turn affects physical health. Accordingly, Hypothesis H2: Physical intention mediates the relationship between physical activity and the health of primary and secondary school teachers is proposed.

Self-efficacy refers to an individual’s level of confidence in his or her ability to perform a given task through his or her own abilities [17], and if an individual’s level of confidence is higher, the higher his or her self-efficacy will be. Research has shown that physical activity can increase an individual’s self-efficacy [18], and that individuals’ participation in physical activity can improve their self-confidence and willpower, which in turn improves their health [19]. Self-efficacy is also an important influence on an individual’s physical health. Self-determination theory suggests that individuals seek autonomy, competence, and relationships in their behavioral choices and persistence [20]. In terms of physical activity, high self-efficacy may be associated with autonomy and motivation for individuals to be more physically active [21]. In health studies of primary and secondary school teachers, individuals’ physical intentions may be closely related to their autonomy and motivation towards health behaviors. Accordingly, hypotheses H3: Self-efficacy mediates the relationship between physical activity and primary and secondary school teachers’ self-assessed health status, and H4: Physical intention and self-efficacy chain mediate the relationship between physical activity and primary and secondary school teachers’ health are proposed.

In summary, the academic community has paid little attention to the health of the primary and secondary school teacher population, with existing research focusing on sociological and medical areas such as burnout, health inequalities, and unhealthy lifestyles, and relatively neglecting the constraints of teachers’ self-efficacy, body intention, and other own psychological factors on their health levels. The study helps to fill the knowledge gap in the existing literature on the relationship between health and physical activity among primary and secondary school teachers, providing a foundation for future research. By gaining a deeper understanding of this relationship, researchers can provide theoretical and empirical support to promote further academic exploration and practice in related fields, thereby advancing the field as a whole. Overall, research on the relationship between physical activity and the health of primary and secondary school teachers can help to promote the health of the teaching population and improve their job satisfaction and overall performance, which in turn will have a positive impact on the development of the education system and students.Therefore, from an active health perspective, this paper explores whether, what kind of, and how physical activity impacts on the health of primary and secondary school teachers, and what role psychological factors such as self-efficacy and body intention play in this to be further recognised.

**Fig. 1:**
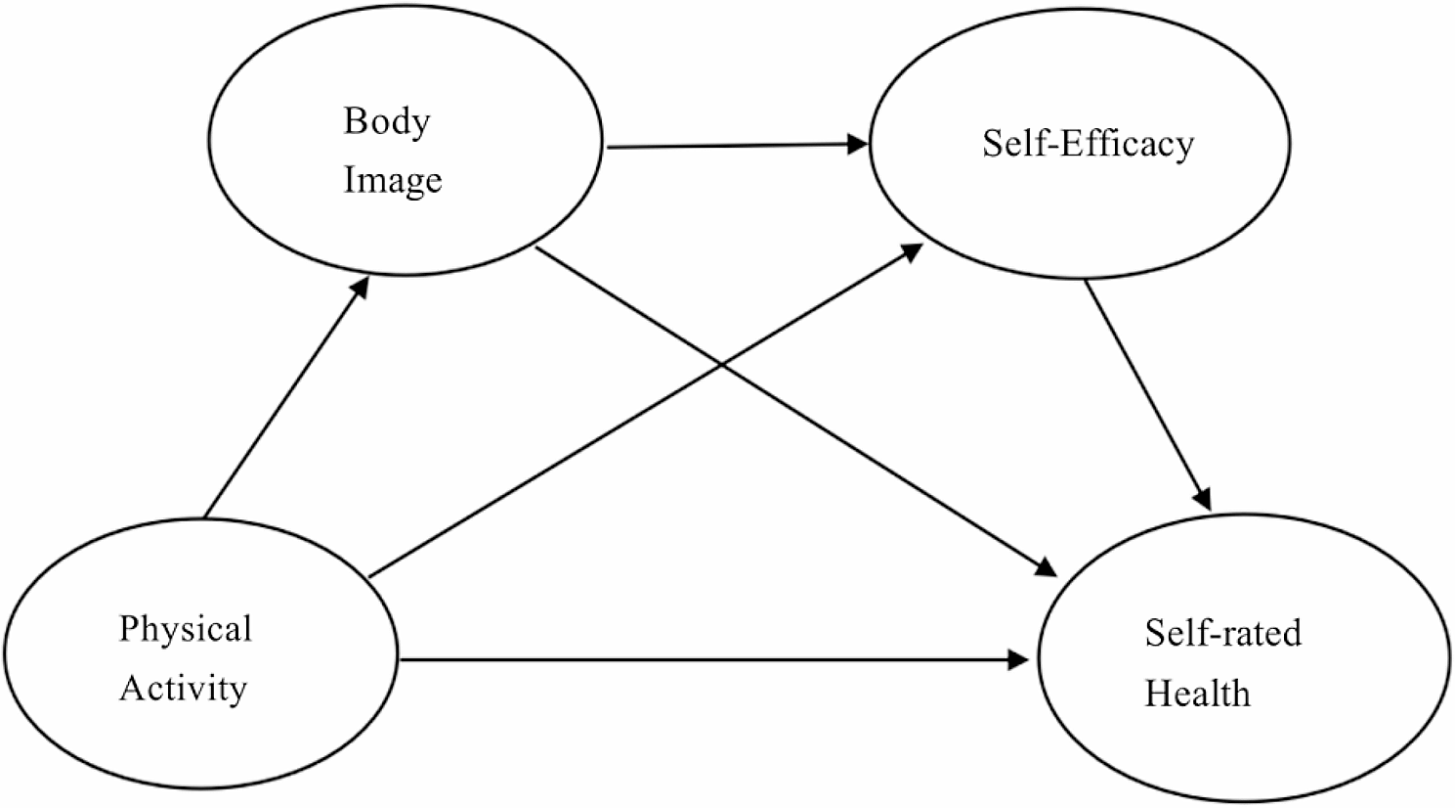
Hypothetical model diagram

### Research design

#### Survey objects

This study conducted a special field research on teachers’ physical and mental health in mainland China from April 2023 to May 2023, using a multi-stage sampling method that included the provincial capitals of 23 provinces and 5 autonomous regions and 4 municipalities directly under the central government in China, and using a random number table method to select 10 cities in each of the capitals of the three provinces in the east, centre and west, as well as in prefectural-level administrative districts, for a total of 30 cities. The quota sampling of the 30 cities was conducted by taking into account the location of schools, school segments, the nature of schools, the scale of schools and the quality of schools, so that the gender, age and urban/rural distribution of the samples obtained basically conformed to the demographic characteristics of the population. More than 120 representatives of primary and secondary school teachers, school headmasters and education administration cadres were summoned to hold seven seminars in order to achieve balanced consideration of teachers’ titles, disciplines, and grades of teaching, a move that helped to ensure the representativeness of the sample selection, and to require that the subjects respond truthfully and carefully in light of the actual situation. After obtaining informed consent from the subjects, the questionnaire was distributed one-on-one and face-to-face with the help of Questionnaire Star, and the respondents answered the questionnaire by clicking on the link online. The subjects were Chinese adult primary and secondary school teachers. Inclusion criteria:①age > 18 years old; ②nationality of the People’s Republic of China;③resident population of China (annual time away from home ≤ 1 month);④voluntary participation in the study and completion of the informed consent form;⑤can complete the online questionnaire by themselves or with the help of the investigator;⑥understand the meaning of the questionnaire expressed in each entry. Exclusion criteria:①people who are delirious or mentally abnormal;②people who are participating in other similar research projects;③people who are unwilling to co-operate.A total of 745 valid questionnaires were produced and collected from teachers of the relevant topics, covering schools of all levels in the basic education sector, both urban and rural. Participants in the research and fill out the questionnaire teachers in line with the objective reality and statistical requirements, through the questionnaire sample distribution characteristics are as follows: 245 males, accounting for 32.89%, 500 females, accounting for 67.11%; age distribution in the 41–50 years old 347 people, accounting for 46.58%, 31–40 years old 182 people, accounting for 24.48%; 21–30 years old 200 people, accounting for 26.85%. There are 200 people aged 21 to 30, accounting for 26.85 per cent of the total. The age ratio of primary and secondary school teachers is good, and there are 111 teachers with the titles of senior and full senior, accounting for 14.90% of the total, and there are 323 first-grade teachers, accounting for 43.36% of the total. There are 373 teachers in the arts category, accounting for 50.07%, 258 in the sciences category, accounting for 34.63%, and 88 in the phonetics, physical education and aesthetics category, accounting for 11.81%, which is a good distribution. The basic demographics are shown in Table 1.

**Table 1:** Demographic characteristic of participants in the study

Variables		Number	Percent	Variables		Number	Percent
Gender	Male	245	32.89%	Job title	Level three	91	12.21%
	Female	500	67.11%		Secondary	220	29.53%
Age	21~30	200	26.85%		level one	323	43.36%
	31~40	182	24.48%		Senior	111	14.90%
	41~50	347	46.58%	Subject classifica-tion	liberal arts	373	50.07%
	≥ 51	129	17.32%		Science	258	34.63%
Teaching grade	primary school	334	44.83%		Music、 sports、art	88	11.81%
	junior high school	210	28.19%		other	26	3.49%
	high school	201	26.98%	number of chronic diseases	no chronic disease	357	47.92%
School area	town	544	73.02%		Suffering from 1 chronic disease	207	27.79%
	rural	201	26.98%		Suffering from 2 or more chronic diseases	181	24.30%

### Measurement tools

#### International physical activity questionnaire short form (IPAQ)

The International Physical Activity Questionnaire (IPAQ) consists of 7 questions, except for the last question that investigates the individual’s sedentary status, the first 6 questions investigate the individual’s physical activity status. Individuals’ physical activity is categorized into 3 categories: low intensity, moderate intensity and high intensity, and the weekly frequency of activities of different intensities and the time of exercise performed per day are recorded. On the scale, the weekly physical activity score = weekly frequency (d/w) * daily exercise time (min\d) * MET assignment, the assignment of walking, moderate-intensity activity, and high-intensity activity were 3.3, 4.0, and 8.0, respectively, and the multiplication score was the individual physical activity level score, which was classified into 3 groups of high, moderate, and low exercise according to the grouping criteria in Table 2.

**Table 2:** Physical activity grouping criteria

Group	Standard
High exercise group	Meet any of the following conditions: 1. Various types of high-intensity physical activity for 3 days or more, and the total weekly physical activity level is greater than or equal to 1500MET-min/w 2. The total of the three intensities of Physical Activity is greater than 7 days, and the total weekly Physical Activity level is greater than or equal to 3000MET-min/w
Moderate exercise group	Meet any of the following conditions: 1. All kinds of high-intensity physical activities of 20 min or more per day, for a total of 3 days or more 2. Various moderate-intensity and/or walking activities of 30 min or more per day for a total of 5 days or more 3. The Physical Activity of 3 intensities totals 5 days or more, and the total weekly Physical Activity level is greater than 600MET-min/w
Low exercise group	Failed to report any exercise, or the exercise level did not meet the medium and high grouping criteria

### Self-assessed health status

This study measured the health status of primary and secondary school teachers through a self-assessed health question. Given that primary and secondary school teachers may strategically underreport or politely overreport their true health status when answering the health questions, and that only 31 people answered “very unhealthy” to this variable in the preliminary stage of data analysis, this study referred to Zhang Wenhong et al.‘s study [21] to reclassify the self-assessed health status of teachers into two levels, namely, “very unhealthy”, “relatively unhealthy”, “very unhealthy”, “relatively unhealthy” and “very unhealthy”, and “relatively unhealthy”. Therefore, in the data analysis, this study referred to Zhang Wenhong et al.‘s study [21], and reclassified teachers’ self-assessed health into two levels, i.e., assigning the answers of “very unhealthy”, “relatively unhealthy” and “average” to 0, which means “unhealthy”; assigning the answers of “very unhealthy”, “relatively unhealthy” and “average” to 0, which means “unhealthy The responses of “very unhealthy”, “quite unhealthy” and “average” were assigned a value of 0 to indicate “unhealthy”, while the responses of “quite healthy” and “very healthy” were assigned a value of 1 to indicate “healthy”.

### Body image states scale (BISS)

The Body Imagery Status Scale (BISS) was developed by Cash et al. [22], and the Chinese version of the scale has been widely used in the measurement of body imagery, with good reliability and validity. The scale consists of 6 entries, which examines the subjects’ perceived status of their physical appearance, body size, weight, attractiveness, self-perception, and comparison with others at a given moment. The scale is scored on a 9-point scale, with 1–9 positive scoring for 1, 3, and 4; and 9 − 1 negative scoring for 2, 4, and 6. The total score of the body image index is the sum of the scores of all the items, and because of the correlation between body imagery and the state in which the respondents are living, neutral scenarios were selected for the respondents in order to safeguard the consistency of the survey results.

### General self-efficacy scale (GSES)

Self-efficacy has a direct impact on an individual’s mental processes when performing an activity, thus playing a decisive role in the choice of human behavior. In this study, we used the GSES, a Chinese version of the General Self-Efficacy Scale (GSES), which was translated and revised by Wang Caikang [23], and which has been widely used in empirical studies at home and abroad. The GSES consists of a total of 10 questions and is designed to be one-dimensional, with a 4-point Likert scale to calculate the scores, with 1 standing for “not at all correct” and 4 stands for “completely correct”. Higher scores indicate higher levels of self-efficacy.

### Reliability test

In this study, the reliability of each scale was tested by the internal consistency coefficient (Cronbach’s α), which is greater than 0.9, indicating a high degree of consistency of the dimensions. Using the orthogonal variance maximization method, the question items of the questionnaire were subjected to the principal component analysis of the factor components, and the KMO statistic was 0.893, which is suitable for the factor analysis, and the sample distribution spherical The significance of Bartlett’s chi-square test is less than 0.001, the total explanation rate is 80.35, the explanation effect is good, the spherical hypothesis is rejected, and the correlation between the original variables indicates that the questionnaire has a high reliability, and then the reliability of the questions of each measurement item, as well as the reliability of the composition of the dimensions and convergent validity of each dimension are further examined through the validation factor analysis, and the results of the validation factor analysis are shown in Table 3, where the standardized factor loadings of the measurement items are shown in Table 3. The standardized factor loadings (STD) of the measurement items are all greater than 0.6, and the path coefficients of the measurement items are all significant (Z > 1.96), indicating that the measurement items have high topic reliability, the squared multiple correlation coefficients (SMC) meet the criterion of being greater than 0.3, and the constitutive reliabilities (CR) are all greater than 0.7, which indicate that the measurement of the latent variables of the indicators conforms to their own qualities, and the average variance extraction (AVE) is greater than 0.7. amount (AVE) is greater than 0.5 (Fornell & Larker) [24], indicating that each dimension has high reliability and convergent validity, the above results show that the results of the validated factor analysis (CFA) calculations support the foregoing, and the results of the exploratory factor analysis (EFA) of all the indexes are better than the recommended values, indicating that all the items of the questionnaire have a certain degree of reliability and validity, and that the scales have a good fit ( See Table 3 for details).

**Table 3:** Internal consistency reliablity coefficients

Variables	(λ)	CR	AVE	Cronbach’s α
Physical Activity	0.779~0.924	0.8771	0.7322	0.809
Body Image	0.872~0.933	0.9363	0.8311	0.935
Self-Efficacy	0.883~0.912	0.9446	0.8295	0.884
Self-rated Health	0.776~0.909	0.9035	0.7241	0.798

### Ethics statement

This study was performed in accordance with the Declaration of Helsinki, and was approved by the Institutional Review Board.

### Mathematical and statistical methods

This study used SPSS 25.0 and Amos 24.0 software for data processing and analysis. Firstly, the valid data were imported into SPSS 26.0 analysis software, and the K-S non-parametric test, reliability analysis, exploratory and validation factor analysis were used to test the normal distribution of the data and the reliability and validity of the instruments. Amos 24.0 was used to validate the scale’s structural validity. And mediated effects model was fitted. Correlation analysis, regression analysis method, and the macro program Process plug-in in SPSS 25.0 were used for the chained mediation effect test and Bootstrap analysis test. The significance level of the statistical results was set at *P* < 0.05.

## Results and analyses

### Common method bias test

Harman’s one-way test was used to conduct an unrotated exploratory factor analysis for all measures. The results showed that a total of four common factors with eigenvalues greater than 1 were proposed, and the first common factor explained 46.69% of the total variance, which was smaller than the judgment criterion of 50% proposed by Podsakoff. It can be assumed that there is no obvious problem of common method bias in this study.

### Descriptive statistical analyses

#### Teachers’ physical activity and health status

According to the findings of the study, teachers do not spend enough time participating in physical activities on a daily basis. Among them, 54.63 per cent of teachers said they never participated in strenuous exercise, and 39.33 per cent did not participate in moderate physical activity; among teachers who participated in physical activity, less than 15 per cent spent an average of about 45–50 min a day and exercised more than three times a week, so the time and intensity of physical exercise were seriously insufficient. In addition, on weekdays, 8.72 per cent of teachers are sedentary for more than 12 h, and only 3.62 per cent are able to control their sedentary time to less than one hour. The above data show that sedentary time and insufficient physical exercise are the main factors affecting teachers’ physical health.

Among the indicators of self-assessed health status, 222 teachers, or 29.8 per cent of the number of teachers surveyed, self-assessed their health status as poor. Of these, 6.98 per cent considered themselves to be “very unhealthy” and 22.82 per cent considered themselves to be “relatively unhealthy”. Another 37.99 per cent of teachers considered themselves to be “relatively healthy” and only 6.04 per cent considered themselves to be “very healthy”. In terms of chronic diseases (such as hypertension, diabetes, high blood cholesterol, etc.), 27.79 per cent of teachers suffer from one chronic disease, 12.89 per cent suffer from two chronic diseases, and 11.41 per cent suffer from three or more, so that, overall, the health of China’s teachers is a cause for concern.

### Teachers’ body intention and self-efficacy status are generally poor

According to the Body Intention Status Scale, in terms of appearance evaluation, 50.33 per cent of the teachers were dissatisfied with their appearance and only 1.74 per cent were extremely satisfied with their appearance. In terms of body part satisfaction, 42.15 per cent of the teachers felt dissatisfied with the size and shape of their body and only 4.3 per cent felt extremely satisfied with the size and shape of their body. In terms of overweight apprehension, 57.86 per cent of the teachers felt dissatisfied with their weight and only 1.21 per cent felt extremely satisfied with their weight. In terms of attitude towards appearance, 30.6% of the teachers felt that they were not physically attractive and only 2.82% felt that they were extremely physically attractive; 42.83% of the teachers felt that they looked worse than usual and only 2.28% felt that they looked great than usual.

As for the statistics of self-efficacy, it was learnt that 53.96% of the teachers had difficulty in sticking to their ideals and reaching their goals, and only 8.59% of them thought it was relatively easy. When facing unexpected situations, 39.86% of the teachers said that they had limited ability to deal with them; 39.6% of the teachers lacked confidence in dealing with problems. In their daily work and life, 39.73 per cent of teachers found it difficult to comfortably cope with any challenges originating from themselves; 39.33 per cent were easily swayed by the opinions of others. Overall, about 31.86 per cent of teachers have a low sense of self-efficacy and poor psychological quality; among them, about 5.62 per cent completely lose confidence in facing difficulties and setbacks, and are unable to cope with difficulties on their own.

### Analysis of variance of self-assessed health status in different physical activity groups

One-way ANOVA was performed with physical activity (low, medium and high) as the independent variable and self-rated health status as the dependent variable respectively (see Table 4). The results showed that the differences between the different physical activity level groups were significant (*p* < 0.001) in the mean scores of self-assessed health status and in all dimensions. The U.S. Department of Health and Human Services and the American Cancer Society recommend that in order to maintain general physical health and prevent chronic diseases, adults need to engage in at least 150 min of moderate-intensity physical activity or 75 min of vigorous-intensity physical activity per week. However, it was found in the course of the survey that less than 15% of teachers who participated in physical activity spent an average of about 45–50 min per day and exercised more than three times per week. Among them, 54.63% of teachers said they never participated in strenuous exercise and 39.33% did not participate in moderate physical activity. The time and intensity of physical exercise of primary and secondary school teachers are seriously insufficient. According to the results of the body image Status Scale, 50.33% of the teachers were dissatisfied with their appearance and only 1.74% were extremely satisfied with their appearance. In terms of body part satisfaction, 42.15% of teachers were dissatisfied with the size and shape of their bodies, while only 4.3% were extremely satisfied with the size and shape of their bodies.

**Table 4:** Descriptive statistics (x ± s) and variance analysis of variables under different groups

	Number	Body Image	Self-Efficacy	Self-rated Health
high exercise group	156	32.04 ± 8.78	29.70 ± 5.65	0.52 ± 0.50
Moderate exercise group	319	29.80 ± 8.01	28.19 ± 5.63	0.33 ± 0.47
low exercise group	270	26.33 ± 8.63	26.03 ± 5.95	0.20 ± 0.40
Physical Activity	745	29.01 ± 8.67	27.72 ± 5.91	0.32 ± 0.47
F		25.261^***^	21.923^***^	24.566^***^
Male	245	28.98 ± 9.00	28.31 ± 6.18	0.33 ± 0.47
Female	500	29.51 ± 8.12	27.69 ± 5.57	0.34 ± 0.47
town	544	29.08 ± 8.41	27.84 ± 5.74	0.33 ± 0.47
rural	201	30.01 ± 8.43	28.03 ± 5.91	0.34 ± 0.47
21~30	200	29.44 ± 9.16	28.63 ± 5.95	0.35 ± 0.48
31~40	182	28.66 ± 8.84	27.58 ± 5.86	0.32 ± 0.48
41~50	347	28.30 ± 8.75	27.22 ± 6.10	0.27 ± 0.45
≥ 51	129	29.93 ± 7.55	27.50 ± 5.55	0.36 ± 0.48
liberal arts	373	28.86 ± 8.59	27.58 ± 5.65	0.28 ± 0.45
Science	258	28.28 ± 8.15	27.20 ± 5.86	0.28 ± 0.45
Music, sports and art	88	31.35 ± 9.99	29.61 ± 6.38	0.56 ± 0.50

*P < 0.05, **P < 0.01, ***P < 0.001

Post hoc tests showed that individuals in the high exercise group were significantly higher (*P* < 0.001) than those in the medium and low exercise groups in the dimensions of body image, self-efficacy, and self-assessed health status, and that individuals in the group that participated in the medium exercise group were significantly higher (*P* < 0.001) than those in the low exercise group in the self-assessed health status and all its dimensions. Independent samples t-test revealed that there was no significant difference in self-efficacy, body image and self-rated health status by gender (*P* > 0.05). There was no significant difference in physical activity by gender (F = 0.151, *P* = 0.698 > 0.05), but the mean value of physical activity for male teachers was 2297.86, which was lower than the mean value of physical activity for female teachers, which was 2456.46. Rural teachers were slightly better than their urban counterparts in scores of Physical Intentions, Self-Efficacy, and Self-assessed Health Condition but none of them were significantly different (*P* > 0.05). In terms of age distribution, teachers aged 41–50 years old have the lowest level of self-assessed health status, which is consistent with the study of JI Ke-Meng [25], and primary and secondary school teachers’ body image and self-assessed health status show a “V” shaped distribution with high at both ends and low in the middle of the age range. In addition, due to the characteristics of the subject, teachers of music, physical education and aesthetics were better than teachers of arts and sciences in all indicators, especially the self-assessed health status indicators.

### Correlation analysis between physical activity, body image, self-efficacy and self-rated health status

Bivariate Pearson’s product difference correlation analyses of the main variables and their dimensions showed that there was a significant positive correlation between physical activity and physical intention, self-efficacy, self-assessed health status and their dimensions; and there was a significant positive correlation between physical intention, self-efficacy and self-assessed health status and their dimensions. There was a positive but not significant correlation between the frequency and duration of physical activity and body image and self-efficacy; the higher the frequency and duration of physical activity, the higher the level of self-rated health status (see Table 5). Descriptive statistics and correlation analyses initially illustrated the relationships between the variables and provided a basis for further data analysis.

**Table 5:** Fornell and Larker method(discriminant validity)

Variables	Physical Activity	Frequency of physical activity	Physical activity time	Body Image	Self-Efficacy	Self-rated Health
Physical Activity	1					
Frequency of physical activity	0.611^**^	1				
Physical activity time	0.565^**^	0.639^**^	1			
Body Image	0.219^**^	0.073	0.082	1		
Self-Efficacy	0.198^**^	0.094	0.112	0.377^**^	1	
Self-rated Health	0.195^**^	0.153^**^	0.201^**^	0.422^**^	0.282^**^	1

*P < 0.05, **P < 0.01, ***P < 0.001

### Regression analysis of physical activity, body image, self-efficacy and self-assessed health status

Multiple linear regression analyses were conducted with self-assessed health status as the dependent variable and physical activity, physical intention and self-efficacy as the independent variables, and the results are shown in Table 6, where the VIF value is less than 5 and the tolerance is greater than 0.2, which indicates that there is no covariance between the variables, where the statistic F = 63.142, *P* < 0.001, so the multiple linear regression passes the test of overall significance and the regression model is statistically significant. The results of the linear regression show that the standardized regression coefficients of physical activity, physical intention and self-efficacy are 0.092, 0.352 and 0.131 respectively; therefore physical intention has the greatest impact on self-assessed health status, followed by self-efficacy and the variables all have a significant positive impact on self-assessed health status.

**Table 6:** Multiple linear regression analysis

Variables	Coefficient	Standard error	Standardized coefficient	t	VIF	Tolerance
Constant	-0.550	0.078	-	-7.017^***^	-	-
Physical Activity	0.001	0.000	0.092	2.728^**^	1.068	0.936
Body Image	0.019	0.002	0.352	9.824^***^	1.196	0.836
Self-Efficacy	0.010	0.003	0.131	3.672^***^	1.185	0.843

*P < 0.05, **P < 0.01, ***P < 0.001

### Mediating role of body image, self-efficacy in physical activity and interpersonal interactions

Based on the correlation analysis, the mechanism of physical activity influencing self-rated health was examined using structural equation modeling, and the constructed model is shown in Fig. 1. The fitting results showed that the model was good for all indicators (χ^2^/df = 4.11, CFI = 0.96, TLI = 0.94, SRMR = 0.03, RMSEA = 0.05). Based on the model fitting results, the chained multiple mediation effects were tested using the bias-corrected nonparametric percentile Bootstrap method, and the study was repeated 5000 times, and the results are shown in Table 7. The results showed that physical activity→physical intention→self-assessed health status, physical activity→self-efficacy→self-assessed health status, physical activity→physical intention→self-efficacy→self-assessed health status. All three mediating effects were significant, as was the total mediating effect, and it can be assumed that the mediating role of physical intention and self-efficacy was established.

**Table 7:** Regression analysis of variable relationships in chain mediation models

Outcome variable	Predictor variable	R Square	F	Standardized β coefficient	t
Self-rated Health	Physical Activity	0.038	29.507^***^		
				0.195	5.432^***^
Body Image	Physical Activity	0.048	37.343^***^		
				0.219	6.111^***^
Self-Efficacy		0.156	68.669^***^		
	Body Image			0.350	10.139^***^
	Physical Activity			0.122	3.516^***^
Self-rated Health		0.204	63.142^***^		
	Physical Activity			0.092	2.728^**^
	Body Image			0.352	9.824^***^
	Self-Efficacy			0.131	3.672^***^

*P < 0.05, **P < 0.01, ***P < 0.001

The mediating path was further tested and the results showed (see Table 8) that the total indirect effect value was 0.0605, 95% CI: 0.138–0.262, indicating that the model with physical intention and self-efficacy as chain mediators was valid. The addition of body intention and self-efficacy resulted in a direct effect of 0.0130 (95% CI: 0.017–0.056), indicating that body intention and self-efficacy act as indirect chain mediators between physical activity and self-assessed health status of primary and secondary school teachers. The total mediating effect (0.0605) accounted for 82.31% of the total effect (0.0735). Specifically, physical activity mainly affects teachers’ self-assessed health status through the following three paths: (1) physical activity→physical intention→self-assessed health status, and the confidence interval of the mediation effect does not contain a value of 0, which indicates that the mediation effect of this path is significant (the value of the indirect effect is 0.0215, accounting for 29.25% of the total effect); (2) physical activity→self-efficacy→self-assessed health status, and the confidence interval of the mediation effect does not contain a value of 0, indicating that the mediation effect of this path is significant. confidence interval does not contain a value of 0, indicating that the mediation effect of this path is significant (the indirect effect value is 0.0200, accounting for 27.21% of the total effect); (3) physical activity → physical intention → self-efficacy → self-assessed health status, the confidence interval of the mediation effect does not contain a value of 0, indicating that the mediation effect of this path is significant (the indirect effect value is 0.0190, accounting for 25.85% of the total effect). The mediation effects of all three pathways reached the significance level.

**Table 8:** Test of the chain mediation effect of body image and self-efficacy on physical activity and self-rated health

Benefit type	Path relationship	Standardized effect size	Boot SE	Bootstrap 95%CI		Effect proportion
				Lower	Upper	
Total effect		0.0735	0.0331	-	-	100%
Direct effect	Physical Activity→Self-rated Health	0.0130	0.0032	0.0172	0.0564	17.69%
Indirect effect 1	Physical Activity→Body Image→Self-rated Health	0.0215	0.0018	0.0179	0.0250	29.25%
Indirect effect 2	Physical Activity→Self-Efficacy→Self-rated Health	0.0200	0.0265	0.0149	0.0252	27.21%
Indirect effect 3	Physical Activity→Body Image→Self-Efficacy→Self-rated Health	0.0190	0.0018	0.0155	0.0225	25.85%
Total indirect effect		0.0605	0.2862	0.1381	0.2619	82.31%

**Fig. 2:**
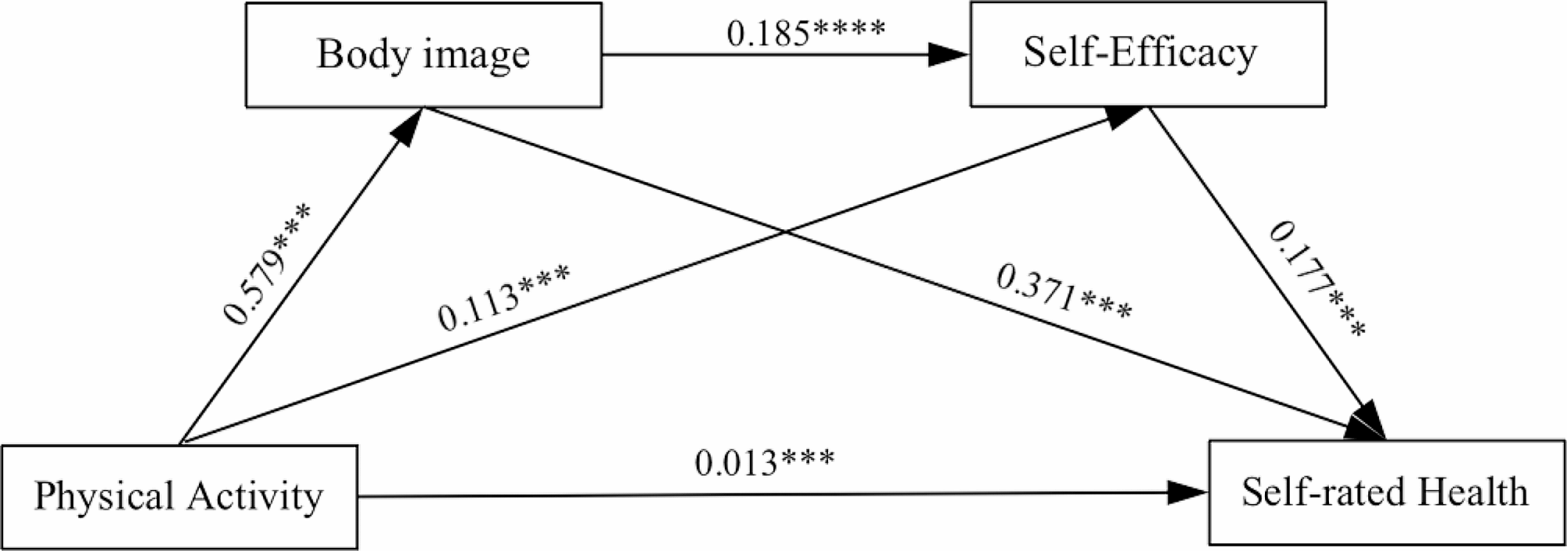
Chained mediation model diagram

Based on the above results, the chain mediation model of physical activity on the self-assessed health status of primary and secondary school teachers was constructed, as shown in Fig. 2.

## Discussion

### Relationship between physical activity and self-assessed health status of primary and secondary school teachers

In the course of this study, it was found that most teachers have a certain degree of health awareness, but their health literacy is not high, and they lack scientific and effective means of fitness. Coupled with the influence of work pressure and family pressure, there is still a great distance from health awareness to health action in the teacher group, resulting in a lack of active health behavior among the majority of teachers and the inability to effectively transmit the concepts of health education to their students. The results of the research show that teachers with poor self-assessed health status accounted for 29.8% of the number of teachers surveyed. Among them, 6.98% of teachers considered themselves “very unhealthy”, 22.82% considered themselves “relatively unhealthy”, 37.99% considered themselves “relatively healthy”, and only 6.04% considered themselves “healthy”. “Only 6.04% of the teachers think they are “very healthy”. The reason for this is that primary and secondary school teachers’ prolonged and intense workloads and excessive classroom hours have led to serious health problems, such as fatigue, lack of stamina and weakened immunity. Teachers’ jobs usually demand a lot of time, which may diminish their opportunities to engage in physical exercise or other physical activities, and their busy working lives may lead to unhealthy dietary choices [26]. In addition, frequent teaching activities, prolonged standing, and exposure to large numbers of students and complex subject matter may increase teachers’ physical load and tension, posing a potential threat to their physical and mental health.

When examining gender differences in physical activity and self-assessed health, it was found that there was no significant difference, while the survey of this study showed that female teachers were more physically active than male teachers, which is inconsistent with previous research [27], most studies believe that men will have greater physical activity than women, the reason for this result may be in psychological factors, women are usually more concerned about their physical appearance and physical health, and therefore are more likely to favor engaging in sport to maintain health and fitness and have higher physical expectations of themselves. In addition, exercise is also seen as an effective way to cope with stress and anxiety, and female teachers may be more inclined to exercise to reduce work and life stress [28]. In the bivariate correlation between frequency and duration of physical activity and self-rated health status, it was concluded that the higher the frequency and duration of physical activity, the higher the self-rated health status scores, specifically, individuals in the high exercise group had significantly higher self-rated health status scores (0.52 ± 0.50) than those in the moderate exercise group (0.33 ± 0.47) and low exercise group (0.20 ± 0.40) (*p* < 0.001), and individuals in the moderate exercise group were significantly higher than those in the low exercise group in terms of self-rated health and its dimensions (*P* < 0.001). This is consistent with the findings of Wenhong Zhang [21] that for every increase in the average number of hours of physical activity per week by teachers, the odds of being healthy increase by 9.09%. The present study found a significant positive correlation (*P* < 0.01) between physical activity and physical health status, confirming that physical activity has a positive promotional effect on the physical health of primary and secondary school teachers, specifically, physical activity enhances physical health in an integrated manner by positively affecting multiple aspects of the cardiovascular system, metabolism, bones, immune system, and mental health. This finding is consistent with previous studies [29] and validates this study’s hypothesis H1. The nature of teachers’ work may lead to a number of physical health problems, but by actively engaging in physical activity, improving their lifestyles, and seeking social support, teachers can better manage these challenges, maintain and improve their health, and thus better fulfill their duties and responsibilities. Governments and school administrations can also take steps to support and promote the health of teachers to ensure that they are able to deliver high-quality educational services.

### Mediating role of body image and self-efficacy in physical activity and self-rated health status

This study not only explored the direct relationship between physical activity and self-rated health status, but also examined the mediating role of body image and self-efficacy between physical activity and self-rated health status. The results showed that physical intention and self-efficacy played a partial mediating role in the effects of physical activity on the self-rated health status of primary and secondary school teachers, and their mediating effects accounted for 29.25% and 27.21% of the total effects, respectively, which verified the hypotheses H2 and H3, and also indicated that physical activity could indirectly improve the self-rated health status of primary and secondary school teachers through improving physical intention and self-efficacy. Among them, physical activity was significantly and positively correlated with both body image and self-efficacy, which is consistent with the results of previous studies [30]. The mediating effect of body image in physical activity and self-rated health has received extensive attention from scholars. Studies have shown that physical activity induces physiological responses, such as improved blood circulation and movement of body muscles, and the intensification of body sensations prompts individuals to pay more attention to and be more sensitive to their own bodies, which in turn enhances body image. Individuals with high body image are usually more attentive to the physical-mental connection, and they are more likely to be physically active, and this positive physical activity can have positive health effects, such as improved cardiovascular health, weight loss, and improved immune system functioning, which in turn improves self-rated health [31]. The tendency to adopt more relaxation and psychoeducation methods to relieve stress and anxiety, along with increased health awareness and promotion of positive health behaviors, leads to improved physical health.

In the study of the mediating role of self-efficacy, Fahmy [32] emphasized that the more care and support an individual receives from the external environment, the greater his sense of self-efficacy, in which participation in physical activity is usually accompanied by social interaction and support; social support refers to understanding, encouragement, and recognition from others, and when an individual is positively supported by friends, family, or coaches, they are more likely to view physical activity as a positive experience and, as a result, have increased self-efficacy. Teachers with high self-efficacy in exercise had more positive emotional experiences, good enjoyment, lower subjective fatigue, and greater enjoyment of exercise, and they were more likely to persist in physical activity because they believed they could overcome obstacles, triumph over difficulties, and succeed. Consistent physical activity improves physical health, including mental health, and thus self-rated health. Thus, the prerequisite for teachers’ self-efficacy improvement lies in favorable external environmental support focusing on the positive subjective construction of teachers’ self-awareness of health [33]. Guidance, assistance and encouragement should be given to teachers for their different needs in order to improve their self-efficacy, create a good health culture, and encourage the whole school staff to participate in physical activities together with students.

### Chain mediating role of physical intention and self-efficacy

According to the mediation effect test, it is confirmed that body image and self-efficacy have a chain mediation role in the chain of influence of physical activity on the self-assessed health status of primary and secondary school teachers, and the total indirect mediation effect value accounts for 82.31%, which verifies Hypothesis H4. This is consistent with the theory of the ABC model of psychology, which on the one hand proves that body image and self-efficacy have a role to play in health status; and on the other hand, it suggests that physical activity can further enhance the health status level of primary and secondary school teachers by enhancing body image and promoting the level of individual self-efficacy. This both enriches the results of existing studies and expands new research areas by further revealing the mechanisms by which physical activity affects body image and self-efficacy according to the model of physical activity and body image and self-efficacy proposed by Sonstron et al. [34]. The model suggests that a person’s body image consists of a number of sub-domains (e.g., physical, cognitive, and social), attractiveness, self-perception, and self-concept of cognitive status in comparison with others [35], and that an individual’s self-efficacy increases with the increase in specific sub-domains body image, and vice versa [36]. Analyzing the reasons behind this, on the one hand, body imagery is the perception and evaluation of one’s own body image, and self-efficacy largely stems from self-perception [37], and higher body imagery leads to positive perceptions and evaluations of the individual’s own self [38], which in turn will enhance self-efficacy. Studies have identified the following main pathways through which body image and self-efficacy as chained mediating variables influence self-rated health: physical activity increases self-efficacy by lowering body BMI [39], helping to create a desirable body shape [40], and enhancing individuals’ positive perceptions of body image [41]. Physical activity has also been used as a post hoc factor in some studies, and body image was found to significantly predict physical activity, i.e., the more positive the body image, the more willing one is to engage in physical activity. As for teachers with low body image, it is likely to originate from their poorer exercise experiences and bad exercise feelings during previous exercise leading to their reluctance to engage in physical activity, and their weak ability to counteract discomfort during exercise, which makes their attitude towards self-efficacy also more negative, and their rejection of physical activity, which makes their self-assessed health status also generally low [42]. Kuwato [43] found that teachers were able to derive positive interpersonal relationships, a higher sense of belonging, and a rich teaching experience from their teaching activities, which enhanced their confidence in their self-body images and boosted their self-efficacy [44]. Teachers with high self-efficacy are not only more optimistic and positive, but also good at utilizing resources and coping with various problems and challenges in teaching with confidence of success, which leads to better work outcomes and healthier bodies [45]. Secondly, better body image can provide positive self-efficacy, protect individuals from negative psychological and emotional influences, and rationalize the negative energy phenomena encountered by teachers in lessons and work, and the maintenance of this benign emotional state ensures good health [46], which suggests that an increase in body image contributes to an increase in the level of health, which occurs through an increase in the level of self-efficacy of the individual. Therefore, the more physical activity a primary and secondary school teacher engages in, the higher the level of body image and the higher the level of self-efficacy, and regular physical activity can gradually contribute to the formation of a psychological virtuous circle, which in turn affects the improvement of physical health. It can be seen that the body image and self-efficacy of primary and secondary school teachers play an important role in the process of physical activity to promote health.

## Conclusions and recommendations

### Conclusion

The results of this study support and expand the viewpoint of exercise for health in the group of primary and secondary school teachers, explore the health promotion status of physical activity and its internal mechanism, have a clearer understanding of the physical intention and self-efficacy of the group of primary and secondary school teachers, and have certain theoretical and practical guidance significance for the improvement of primary and secondary school teachers’ physical health, which provides a reference for better attention to the physical health of primary and secondary school teachers. The main conclusions of the study are as follows. The main findings of the study are as follows:

(1) Teachers’ health is poor, and there are problems such as insufficient awareness of teachers’ health, insufficient institutional protection, insufficient supply of health services, and insufficient amount of physical activity.

(2) Physical activities of different activity levels have a promoting effect on teachers’ physical health, and the higher the activity frequency and the higher the activity level, the better the promoting effect.

(3) Body image and self-efficacy play a partial mediating role between physical activity and the health status of primary and secondary school teachers, i.e., physical activity not only directly and positively promotes the physical health of primary and secondary school teachers, but also further affects physical health through the chain mediating role of body image and self-efficacy.

### Recommendations

#### Improve quality and promote the concept of active health for teachers

The multidimensional functions of physical activity in health promotion and active health should be widely disseminated to draw the attention of society to teachers’ health. Firstly, to promote the “Teachers’ Health Action Plan”, in 2018, the Ministry of Education launched the “Teachers and Students Healthy China Health” theme of health education activities, which has had a great impact on social publicity, but the focus of the activities is mainly on students, and can be further optimized in the future. In the future, it is possible to further optimize the form of the campaign, taking teachers as an important activity group and raising their awareness of active health. It is recommended that the education administration department take the lead, collaborate with the publicity department to increase the publicity of health knowledge, select typical practices of physical exercise for teaching staff, disseminate good voices and stories of health on campus, and create a good atmosphere of active health culture. Secondly, we should play the leading role of the physical education teacher group, with the help of carrying out the physical activity time, encouraging the whole school staff and students to participate in physical exercise, and comprehensively leading the school staff to participate in the atmosphere of physical exercise.

### Empowerment and strengthening of the health system for teachers

First, strengthen physical fitness testing and fitness guidance for teachers. Relevant guiding policies have been formulated to regularly carry out physical fitness tests for the teachers’ community, incorporating them into the national physical fitness test program, so as to get an overall grasp of the physical quality and health level of the teachers’ community, and to introduce timely and targeted fitness guidance for teachers, so as to effectively improve teachers’ physical fitness and health. Secondly, improve the funding system for teachers’ physical examinations. Physical examination as an effective segment of disease screening, is the most basic health protection of school teachers, and the current teacher physical examination funds mostly from the school education public funds, funding constraints. Therefore, there is a need to improve the funding system for teachers’ physical examinations, explore the setting up of special funding for teachers’ physical examinations, strengthen the system of regular physical examinations, and increase the breakdown of physical examinations around occupational diseases arising from teachers’ work. Make teachers’ physical health an important part of the work of education administrations at all levels.

### Reduce and increase the burden and optimise the supply of health services for teachers

Firstly, teachers’ workload should be reduced and time for physical activity should be guaranteed. Teachers’ perception of workload is the key to self-efficacy and professional well-being. Excessive workload not only affects physical and mental health, but also reduces teachers’ enthusiasm for teaching. Therefore, the workload of teachers should be reduced by lowering the stock of work, avoiding excessive educational inspections, administrative supervision and other tasks, and exploring different forms of flexible commuting systems for teachers. Secondly, attention should be paid to the physical activity needs of different teachers. Establishing a sound mechanism for classifying and managing teachers’ health, making precise interventions for teachers’ health according to their different career periods and specialties, and formulating physical activity guidance programs. Thirdly, building comprehensive health centers for teachers and students. In view of the poor health status of the teachers’ group and the general lack of knowledge related to scientific physical exercise, it is recommended that each school play the role of trade unions, actively organize training for teachers in fitness and sports knowledge and physical skills, and advocate the construction of an integrated health center in each school, so that the majority of teachers can carry out health monitoring and proactive interventions in school, help teachers with a large daily workload to carry out physical assessment and scientific exercise, and make the school the most convenient fitness place for teachers. the most convenient fitness place for teachers.

### Electronic supplementary material

Below is the link to the electronic supplementary material.

Supplementary Material 1

Supplementary Material 2

## Data Availability

The datasets used and/or analysed during the current study available from the corresponding author on reasonable request.
