# Supplementary material for: The relationship between physical activity and the health of primary and secondary school teachers: the chain mediating effects of body image and self-efficacy
Source: BMC Public Health. 2024 Feb 22;24:562. doi: 10.1186/s12889-024-17914-2 (PMC10882871; doi:10.1186/s12889-024-17914-2)
Supplement: Supplementary file 1 — Supplementary Material 1 [file 12889_2024_17914_MOESM1_ESM.pdf]

A study on the relationship between physical activity and self-rated health status of primary and secondary school teachers

Dear teacher:

Hello! Thank you for participating in this survey. This survey is anonymous and your answers will be kept strictly confidential and will be used for academic research only. There are no right or wrong, good or bad answers to this questionnaire. Please answer according to the actual situation. Thank you again for your support of our research work. I wish you success in your work and a happy life!

1. SEX： [Single choice question] *

○Male

○Female

2. AGE [Single choice question] *

○18

○19

○20

○21

○22

○23

○24

○25

○26

○27

○28

○29

○30

○31

○32

○33

○34

○35

○36

○37

○38

○39

○40

○41

○42

○43

○44

○45

○46

○47

○48

○49

○50

○51

○52

○53

○54

○55

○56

○57

○58

○59

○60

○61

○62

○63

○64

○65

○66

○67

○68

○69

○70

3. Your teacher title: [Single choice question]*

○Level 3

○Level 2

○First level

○Advanced

○High quality

4. Your teaching field: [Single choice question] *

○Liberal Arts

○Science

○Music, sports and beauty

○Other categories

5. Your height: [Single choice question] *

○140cm

○141cm

○142cm

○143cm

○144cm

○145cm

○146cm

○147cm

○148cm

○149cm

○150cm

○151cm

○152cm

○153cm

○154cm

○155cm

○156cm

○157cm

○158cm

○159cm

○160cm

○161cm

○162cm

○163cm

○164cm

○165cm

○166cm

○167cm

○168cm

○169cm

○170cm

○171cm

○172cm

○173cm

○174cm

○175cm

○176cm

○177cm

○178cm

○179cm

○180cm

○181cm

○182cm

○183cm

○184cm

○185cm

○186cm

○187cm

○188cm

○189cm

○190cm

○191cm

○192cm

○193cm

○194cm

○195cm

○196cm

○197cm

○198cm

○199cm

○200cm

6. Your weight: [Single choice question] *

○30kg

○31kg

○32kg

○33kg

○34kg

○35kg

○36kg

○37kg

○38kg

○39kg

○40kg

○41kg

○42kg

○43kg

○44kg

○45kg

○46kg

○47kg

○48kg

○49kg

○50kg

○51kg

○52kg

○53kg

○54kg

○55kg

○56kg

○57kg

○58kg

○59kg

○60kg

○61kg

○62kg

○63kg

○64kg

○65kg

○66kg

○67kg

○68kg

○69kg

○70kg

○71kg

○72kg

○73kg

○74kg

○75kg

○76kg

○77kg

○78kg

○79kg

○80kg

○81kg

○82kg

○83kg

○84kg

○85kg

○86kg

○87kg

○88kg

○89kg

○90kg

○91kg

○92kg

○93kg

○94kg

○95kg

○96kg

○97kg

○98kg

○99kg

○100kg

7. What is your teaching level: [Single choice question] *
○Elementary school
○Junior high school
○High school

8. Is the school you teach in an urban or rural school: [Single-choice question] *
○Urban school
○Rural school

9. Considering your age, how do you think your physical health is: [Single-choice question] *
○Very unhealthy
○Relatively unhealthy
○General health
○Relatively healthy
○very healthy

10. Number of chronic diseases you suffer from: [Single choice question] *

(Such as diabetes, coronary heart disease, hypertension, dyslipidemia, obesity, cancer, asthma, arthritis, allergies, sinusitis, heart failure, chronic obstructive pulmonary disease, chronic kidney disease, depression, back pain, etc.)

○No chronic diseases

○Suffering from 1 chronic disease

○Suffering from 2 chronic diseases

○Suffering from 3 or more chronic diseases

11. Do you have the habit of smoking and drinking: [Single choice question] *

○No smoking or drinking

○Only smoke and don’t drink alcohol

○No smoking, only drinking

○Smoking and drinking

Part 2 IPAQ Short Questionnaire

The following question is to investigate your physical activity during extracurricular time in the past week. Please choose the option that is most appropriate for you. There is no right or wrong answer.

12. In the past 7 days, how many days have you done strenuous physical activities, such as lifting heavy objects, digging, aerobic exercise, or riding a bicycle quickly? [Single choice question] *

○0 (Please skip to question 14)

○1

○2

○3

○4

○5

○6

○7

13. How much time do you usually spend on strenuous physical activities on this day [Matrix text question] [Enter a number from 10 to 240] *

Time (minute)	________________

14. In the past 7 days, how many days have you done moderate physical activity, such as lifting light objects, riding a bicycle at a normal speed, or playing doubles tennis? Please do not include walking. [Single choice question] *

○0 (Please skip to question 16)

○1

○2

○3

○4

○5

○6

○7

15. How much time do you usually spend on moderate physical activity on this day [Matrix text question] [Enter a number from 10 to 240] *

Time (minute)	________________

16. In the last 7 days, how many days did you walk for at least 10 minutes at a time? [Single choice question] *

○0 (Please skip to question 18)

○1

○2

○3

○4

○5

○6

○7

17. How much time do you usually spend walking on this day [matrix text question] [enter a number from 10 to 240]*

Time (minute)	______________

18. In the past 7 days, how much time have you spent sitting down during workdays [Matrix single-choice question]*

	Less than 1 hour	2	3	4	5	6	7	8	9	10	11	more than 12 hours

Time（hours）	○	○	○	○	○	○	○	○	○	○	○	○

Part 3 Body Intentional State Scale

Please read the following paragraph of material and answer the following six questions according to your feelings after reading. Please choose the option that suits you. There is no right or wrong answer. You don't need to think too much, just rely on your first impression. You can only choose one answer for each question, be careful not to miss any question.
Please answer based on how you feel now:

19. Now I feel... [Single choice question] *
○ Extremely dissatisfied with my physical appearance
○Very dissatisfied with my body appearance
○Basically dissatisfied with my body appearance
○A little dissatisfied with the appearance of my body
○No comments about my physical appearance
○Somewhat satisfied with my physical appearance
○Basically satisfied with my physical appearance
○Very satisfied with my physical appearance
○Extremely satisfied with my physical appearance

20. Now I feel... [Single choice question] *
○Extremely satisfied with my body size and shape
○Very satisfied with my body size and shape
○Basically satisfied with my body size and appearance
○Somewhat satisfied with my body size and shape
○No comments about the size or shape of my body
○A little dissatisfied with the size and shape of my body
○Basically dissatisfied with my body size and appearance
○Very dissatisfied with my body size and appearance
○Extremely dissatisfied with my body size and appearance

21. Now I feel... [Single choice question] *

○ Extremely dissatisfied with my weight
○Very dissatisfied with my weight
○Basically not satisfied with my weight
○A little dissatisfied with my weight
○No comments about my weight
○A little satisfied with my weight
○Basically satisfied with my weight
○Very satisfied with my weight
○Extremely satisfied with my weight

22. Now I feel... [Single choice question] *
○My body is extremely attractive
○My body is more attractive
○My body is generally attractive
○My body is slightly attractive
○No comment on my physical attractiveness
○I am somewhat unattractive physically
○I am basically not physically attractive
○I am very unattractive physically
○I am extremely unattractive physically
23. Now I feel... [Single choice question] *
○My appearance is much worse than usual
○My appearance is much worse than usual
○My appearance is worse than usual
○My appearance is only slightly worse than usual
○My appearance is the same as usual
○My appearance is only slightly better than usual
○My appearance is better than usual
○My appearance is much better than usual
○I look better than usual

24. Now I feel... [Single choice question] *
○Far more beautiful than the average person
○Much better looking than ordinary people
○Better looking than average people
○Only slightly prettier than the average person
○ Same as ordinary people
○Only slightly uglier than the average person
○A bit uglier than the average person
○ Much uglier than the average person
○Far uglier than the average person

Part 4 General Self-Efficacy Scale

25. Read the following questions carefully and choose the one that best suits you. There is no right or wrong here, please answer truthfully according to your own situation. [Matrix multiple choice questions] *

	Not at all consistent	Somewhat inconsistent	Somewhat consistent	Completely consistent
I can always solve problems if I try my best	○	○	○	○
Even if others are against me, I still have a way to get what I want	○	○	○	○
It’s easy for me to stick to my ideals and achieve	○	○	○	○

my goals				
I am confident that I can effectively deal with any unexpected things	○	○	○	○
With my intelligence, I will be able to cope with unexpected situations	○	○	○	○
I can solve most problems if I put in the necessary effort	○	○	○	○
I can face difficulties calmly because I trust my	○	○	○	○

ability to deal with problems				
When faced with a problem, I can usually find several solutions	○	○	○	○
When I'm in trouble, I can usually think of some ways to deal with it	○	○	○	○
No matter what happens to me, I can handle it	○	○	○	○
