# Supplementary material for: The relationship between physical activity and the health of primary and secondary school teachers: the chain mediating effects of body image and self-efficacy
Source: BMC Public Health. 2024 Feb 22;24:562. doi: 10.1186/s12889-024-17914-2 (PMC10882871; doi:10.1186/s12889-024-17914-2)
Supplement: Supplementary file 2 — Supplementary Material 2 [file 12889_2024_17914_MOESM2_ESM.pdf]

Checklist for Reporting Of Survey Studies (CROSS)

Section/topic	Item	Item description	Reported on page #
**Title and abstract**			**1**
Title and abstract	1a	State the word "survey" along with a commonly used term in title or abstract to introduce the study's design.	
	1b	Provide an informative summary in the abstract, covering background, objectives, methods, findings/results, interpretation/discussion, and conclusions.	1
**Introduction**			**2**
Background	2	Provide a background about the rationale of study, what has been previously done, and why this survey is needed.	2
Purpose/aim	3	Identify specific purposes, aims, goals, or objectives of the study.	4
**Methods**			**6**
Study design	4	Specify the study design in the methods section with a commonly used term (e.g., cross-sectional or longitudinal).	5
Data collection methods	5a	Describe the questionnaire (e.g., number of sections, number of questions, number and names of instruments used).	5
	5b	Describe all questionnaire instruments that were used in the survey to measure particular concepts. Report target population, reported validity and reliability information, scoring/classification procedure, and reference links (if any).	5
	5c	Provide information on pretesting of the questionnaire, if performed (in the article or in an online supplement). Report the method of pretesting, number of times questionnaire was pre-tested, number and demographics of participants used for pretesting, and the level of similarity of demographics between pre-testing participants and sample population.	5
	5d	Questionnaire if possible, should be fully provided (in the article, or as appendices or as an online supplement).	5
Sample characteristics	6a	Describe the study population (i.e., background, locations, eligibility criteria for participant inclusion in survey, exclusion criteria).	6
	6b	Describe the sampling techniques used (e.g., single stage or multistage sampling, simple random sampling, stratified sampling, cluster sampling, convenience sampling). Specify the locations of sample participants whenever clustered sampling was applied.	6
	6c	Provide information on sample size, along with details of sample size calculation.	6
	6d	Describe how representative the sample is of the study population (or target	6

		population if possible), particularly for population-based surveys.	
Survey administration	7a	Provide information on modes of questionnaire administration, including the type and number of contacts, the location where the survey was conducted (e.g., outpatient room or by use of online tools, such as SurveyMonkey).	5
	7b	Provide information of survey's time frame, such as periods of recruitment, exposure, and follow-up days.	5
	7c	Provide information on the entry process:<br>–>For non-web-based surveys, provide approaches to minimize human error in data entry.<br>–>For web-based surveys, provide approaches to prevent "multiple participation" of participants.	5
Study preparation	8	Describe any preparation process before conducting the survey (e.g., interviewers' training process, advertising the survey).	5
Ethical considerations	9a	Provide information on ethical approval for the survey if obtained, including informed consent, institutional review board [IRB] approval, Helsinki declaration, and good clinical practice [GCP] declaration (as appropriate).	9
	9b	Provide information about survey anonymity and confidentiality and describe what mechanisms were used to protect unauthorized access.	9
Statistical analysis	10a	Describe statistical methods and analytical approach. Report the statistical software that was used for data analysis.	9
	10b	Report any modification of variables used in the analysis, along with reference (if available).	9
	10c	Report details about how missing data was handled. Include rate of missing items, missing data mechanism (i.e., missing completely at random [MCAR], missing at random [MAR] or missing not at random [MNAR]) and methods used to deal with missing data (e.g., multiple imputation).	9
	10d	State how non-response error was addressed.	9
	10e	For longitudinal surveys, state how loss to follow-up was addressed.	9
	10f	Indicate whether any methods such as weighting of items or propensity scores have been used to adjust for non-representativeness of the sample.	9
	10g	Describe any sensitivity analysis conducted.	9
**Results**			
Respondent characteristics	11a	Report numbers of individuals at each stage of the study. Consider using a flow diagram, if possible.	6
	11b	Provide reasons for non-participation at each stage, if possible.	6

	11c	Report response rate, present the definition of response rate or the formula used to calculate response rate.	6
	11d	Provide information to define how unique visitors are determined. Report number of unique visitors along with relevant proportions (e.g., view proportion, participation proportion, completion proportion).	6
Descriptive results	12	Provide characteristics of study participants, as well as information on potential confounders and assessed outcomes.	6
Main findings	13a	Give unadjusted estimates and, if applicable, confounder-adjusted estimates along with 95% confidence intervals and p-values.	9
	13b	For multivariable analysis, provide information on the model building process, model fit statistics, and model assumptions (as appropriate).	11
	13c	Provide details about any sensitivity analysis performed. If there are considerable amount of missing data, report sensitivity analyses comparing the results of complete cases with that of the imputed dataset (if possible).	15
**Discussion**			
Limitations	14	Discuss the limitations of the study, considering sources of potential biases and imprecisions, such as non-representativeness of sample, study design, important uncontrolled confounders.	16
Interpretations	15	Give a cautious overall interpretation of results, based on potential biases and imprecisions and suggest areas for future research.	17
Generalizability	16	Discuss the external validity of the results.	18
**Other sections**			
Role of funding source	17	State whether any funding organization has had any roles in the survey's design, implementation, and analysis.	21
Conflict of interest	18	Declare any potential conflict of interest.	21
Acknowledgements	19	Provide names of organizations/persons that are acknowledged along with their contribution to the research.	21
